# Late deep cervical infection after anterior cervical discectomy and fusion: a case report and literature review

**DOI:** 10.1186/s12891-019-2783-x

**Published:** 2019-09-25

**Authors:** Ying-Chun Chen, Lin Zhang, Er-Nan Li, Li-Xiang Ding, Gen-Ai Zhang, Yu Hou, Wei Yuan

**Affiliations:** Department of Spine Surgery, Beijing Shijitan Hospital, Capital Medical University, No.10 Tieyi Road, Yangfangdian, Beijing, 100038 China

**Keywords:** Deep cervical abscess, Late infection, ACDF

## Abstract

**Background:**

Anterior cervical discectomy and fusion (ACDF) is often performed for the treatment of degenerative cervical spine. While this procedure is highly successful, 0.1–1.6% of early and late postoperative infection have been reported although the rate of late infection is very low.

**Case presentation:**

Here, we report a case of 59-year-old male patient who developed deep cervical abscess 30 days after anterior cervical discectomy and titanium cage bone graft fusion (autologous bone) at C3/4 and C4/5. The patient did not have esophageal perforation. The abscess was managed through radical neck dissection approach with repated washing and removal of the titanium implant. *Staphylococcus aureus* was positively cultured from the abscess drainage, for which appropriate antibiotics including cefoxitin, vancomycin, levofloxacin, and cefoperazone were administered postoperatively. In addition, an external Hallo frame was used to support unstable cervical spine. The patient’s deep cervical infection was healed 3 months after debridement and antibiotic administration. His cervial spine was stablized 11 months after the surgery with support of external Hallo Frame.

**Conclusions:**

This case suggested that deep cervical infection should be considered if a patient had history of ACDF even in the absence of esophageal perforation.

## Background

Anterior cervical discectomy and fusion (ACDF) procedure has been widely performed for degenerative disc disease, traumatic cervical diseases, or cervical spondylosis [1–4]. In a retrospective study, it has been reported that overall morbidity rate of adverse events after ACDF was 19.3% and mortality rate was 0.1% [5]. The most common complication was postoperative dysphagia (9.5%) followed by postoperative hematoma (5.6%), symptomatic recurrent laryngeal nerve palsy (3.1%), dural penetration (0.5%), esophageal perforation (0.3%), worsening of preexisting myelopathy (0.2%), Horner’s syndrome (0.1%), instrumentation back-out (0.1%), and superficial wound infection (0.1%).

Literature search indicated that overall wound infection rate following ACDF is very low with a range of 0.1–1.6% [5, 6], and most infections occur in the early postoperative period as a result of intraoperative bacterial seeding or postoperative poor wound care [7]. However, late infections may also occur [7–9], which are mainly associated with an esophageal perforation [8, 10–13], and occasionally associated with implant migration [6, 10], Zenker’s diverticulum [14, 15], or bacterial seeding from other surgical site or bacteremia [16, 17].

Late infection after an interval of time after ACDF may present with various symptoms and signs including neck pain, dysphagia, and fever accompanied with laboratory abnormalities such as elevated white blood cell count, ESR, and CRP [7, 17]. Neck plain X-ray, CT, MRI, and barium-swallow could be performed to diagnose or exclude esophageal perforation. Bacterial cultures from the wound infection could be performed to identify pathogens and to use appropriate antibiotics.

Here, we report a case of 59-year-old male patient who developed a deep cervical abscess 30 days after ACDF surgery. Clinical and laboratory examination showed evidence of anterior cervical deep abscess with vertebral body movement. A radical debridement, removal of infected implant, and repeated washout of the abscess cavity were performed. An external Hallo frame was applied to support unstable cervical spine in addition to administration of culture sensitive antibiotics to the organism of *Staphylococcus aureus*.

## Case presentation

A 59-year-old man was hospitalized on November 25th, 2013 with the diagnosis of cervical spondylosis. On November 28th, 2013, the patient received anterior cervical discectomy and titanium cage bone graft fusion (autologous bone) at C3/4 and C4/5. The surgery was smoothly performed and a draining tube was placed. Drained volume was 20 mL on day one and 5 mL on day two after the operation, and the draining tube was removed 48 h after operation. After the surgery, patient had methylprednisolone (80 mg, IV once a day for 3 days) and cefotiam (1 g, IV twice daily for 4 days). The patient was discharged from the hospital 11 days after the ACDF.

**Fig. 1:**
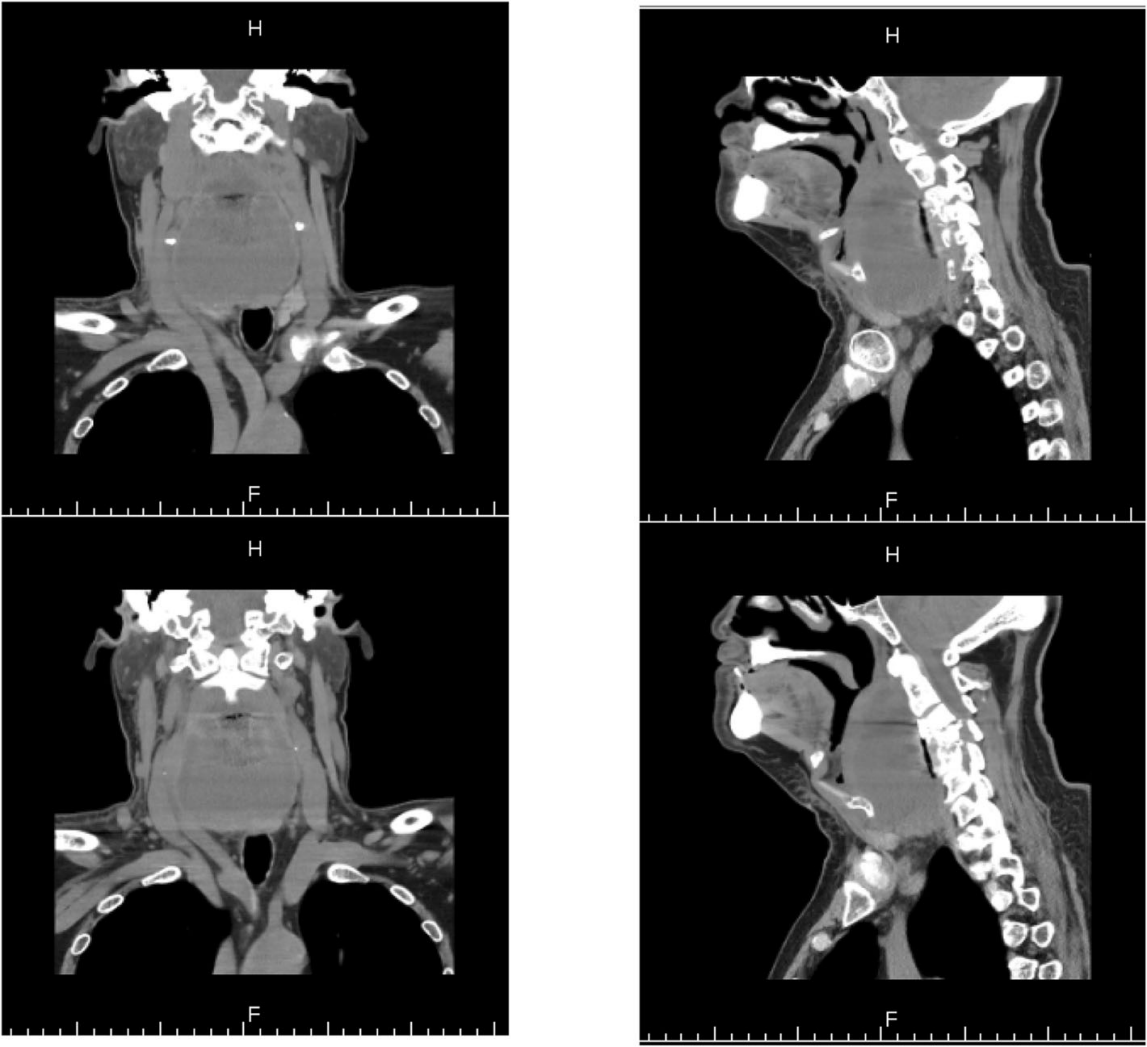
CT scan images on admission

**Fig. 2:**
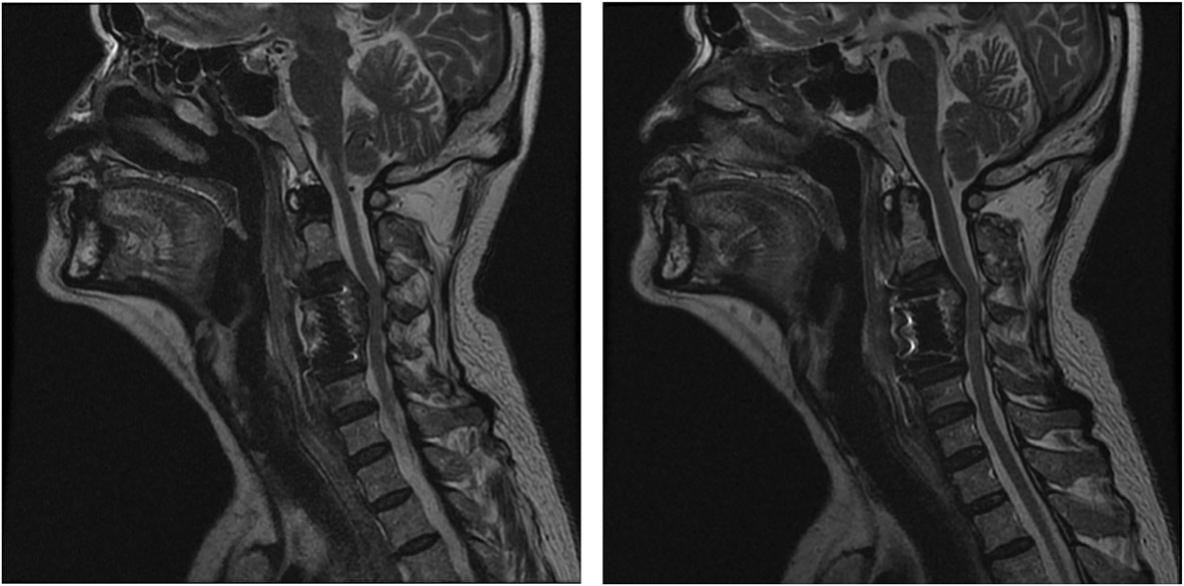
MRI images on admission

**Fig. 3:**
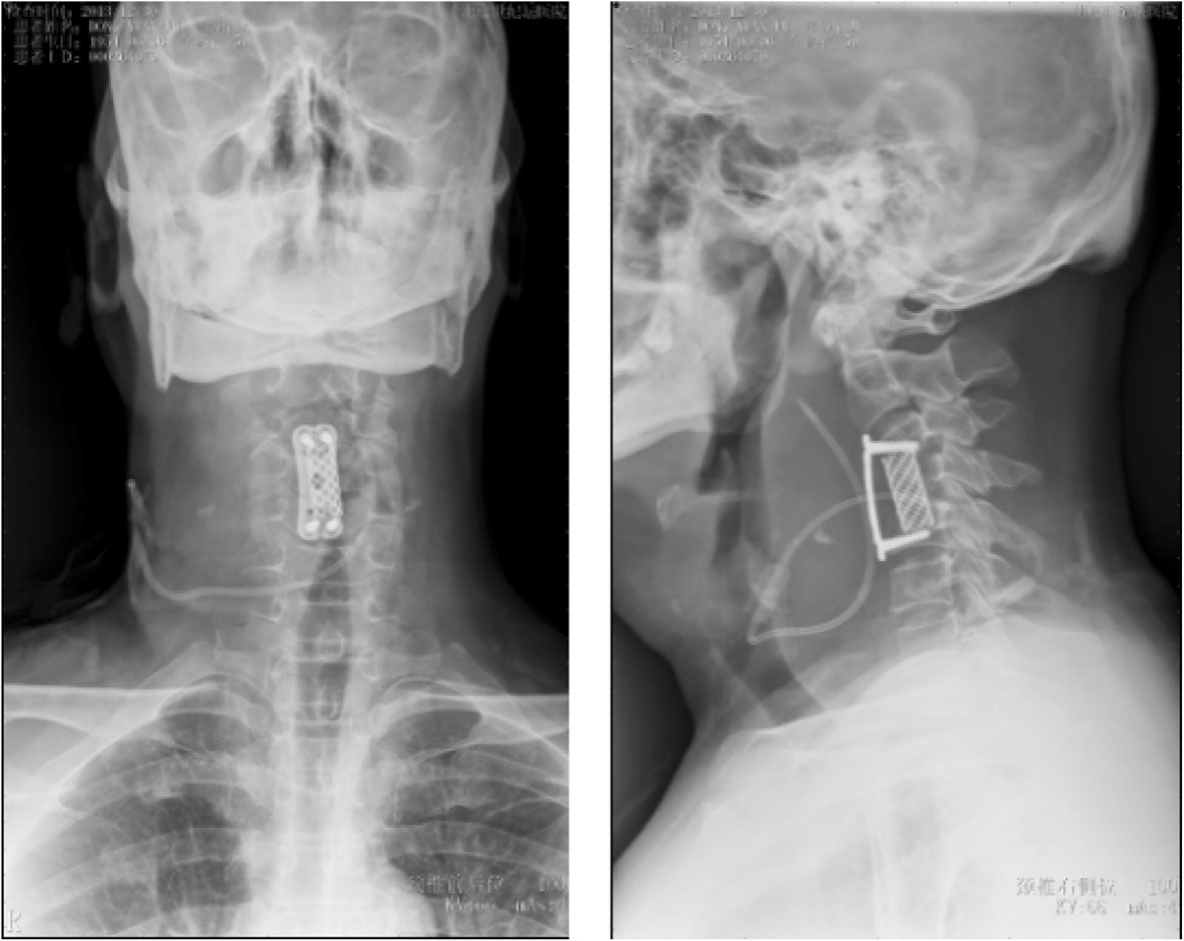
Plain radiograph of neck and implant on admission

On December 30th, 2013 (over one month after the ACDF), the patient came to the emergency room with dysphagia and dyspnea for 1 day as well as an erosion at his anterior neck for 5 days. Physical examination showed body temperature was 36.5 °C, pulse 78 beat/min. Postoperative scar (10 cm length) on the anterior neck and a touchable erosion on right anterior neck were found. Skin color was normal and no pain on press of the erosion was noticed. Computerize tomography (CT) scan (Fig. 1) and magnetic resonance imaging (MRI, Fig. 2) indicated a cystic low-density shadow (36HU) with a size of 69 × 46 mm and smooth wall, which located from C2-C7. Neck plain X-ray showed C3-C5 internal fixation loosed, C3/4 vertebrate moved backward, anterior space of C3-C5 was unclear, and anterior cervical tissue swelled with 4.4 cm thickness by 15 cm length (Fig. 3). Laboratory tests: WBC 12.23 ×  10^9^, NE 81.1%, Hb 104, ESR 88 mm/h, C-reactive protein 104.51 mg/L; prothrombin time 14.4 s, prothrombin activity 57%, prothrombin international normalized ratio 1.32.

**Fig. 4:**
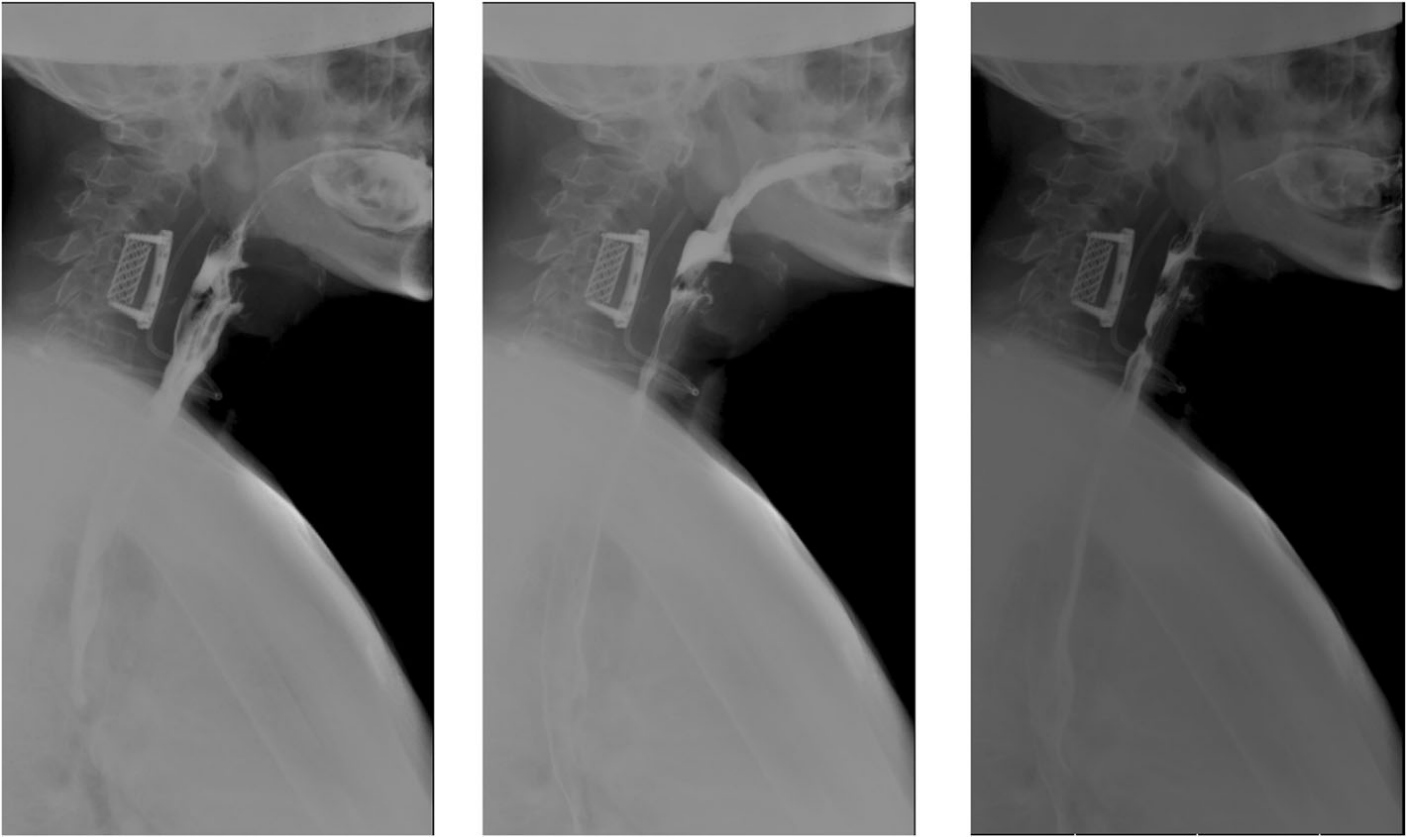
Barium-swallow radiograph

On December 30th, under the guidance of ultrasonography, abscess puncture was performed and 20 ml purulent liquid was extracted from the abscess, and a drainage tube was then placed. Additional 15 mL pink-milky liquid was extracted on December 31st, 2013; 50 mL on January 1st, 2014; 100 mL on January 2nd; and 15 mL on January 3rd. Esophageal perforation was not found by barium-swallow radiography on January 2nd, 2014 (Fig. 4).

*Staphylococcus aureus* was positively cultured from the abscess drainage fluid and antibiotic sensitivity/resistance test indicated that it was resistant to penicillin G, clindamycin, erythromycin, and rifampin, but sensitive to ß-lactamase, cefoxitin, ciprofloxacin, gentamycin, vancomycin, ntrofurantoin, and levofloxacin. Therefore, the patient was given cefotiam (1.0 Bid IV) from December 30th, 2013 through January 1st, 2014 followed by switching to vancomycin (1000 mg, Bid IV) and levofloxacin (0.2 Bid IV) on January 3rd, 2014.

**Fig. 5:**
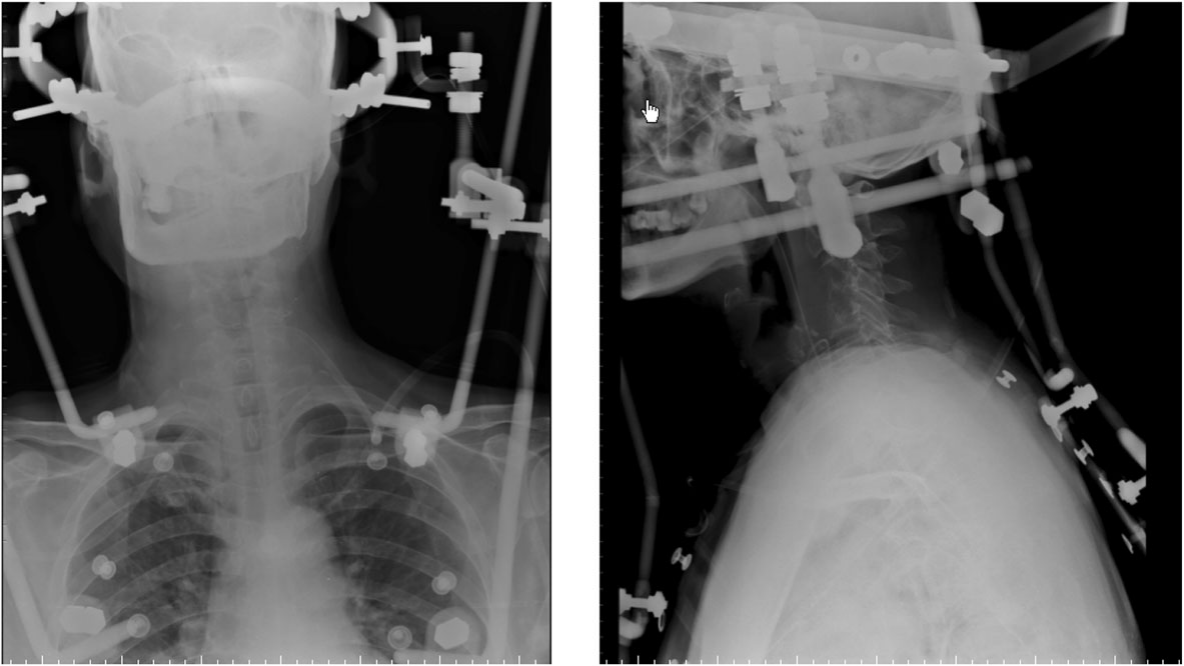
Plain radiograph after application of Hallo frame support

On January 6th, 2014, under systemic anesthesia, the titanium net cage was removed and a radical debridement was performed followed by washing the abscess cavity with saline containing diluted iodophor and amikacin. A tube was placed and the abscess cavity was continuously washed with 500 mL saline containing amikacin (0.2) three times a day from January 6th through 30th, 2014. On January 7th, 2014, an external Hallo frame support was applied (Fig. 5). Vacomycin and levofloxacin were continuously used from January 6th though 20th, 2014.

Laboratory tests indicated that white blood cell count and neutrophil were significantly increased on the day of admission (December 30th, 2013) through January 10th, 2014 (Table 1), which gradually dropped to normal from January 13th to 18th. On January 20th, 2014, patient had high fever (39.3 °C) without abnormal appearance of chest X-ray or laboratory test (Table 1), and antibiotics were changed from levofloxacin to cefoperazone. On January 22nd, 2014, patient still had high fever (39.1 °C) although the laboratory test showed normal white blood cell count and neutrophil percentage (Table 1) and CT scan of neck, chest and abdomen showed no infection. Therefore, vancomycin and cefoperazone were stopped, and avelox (moxifloacin hydrochloride) was initiated after consulting with a Physician of The Department of Infection. Next day, body temperature dropped to 37.1 °C and bacterial cultures were negative from the blood samples from January 20 to 22, 2014 as well as wound washing solution from January 27 to 29, 2014. The patient was discharged from hospital on February 7th, 2014 with normal results of laboratory tests (Table 1).

**Table 1:** Results of laboratory tests

Date	WBC (× 10^9^)	NE (%)	Hb	ESR (mm/h)	CRP (mg/L)
Dec 30th, 2013	12.23	81.1	104	88	104.51
Jan 10th, 2014	13.53	84.3	102	66	33.7
Jan 13th, 2014	7.81	76	89	88	29.74
Jan 18th, 2014	5.84	72.7	93	65	24.52
Jan 20th, 2014	4.62	56.1	94		
Jan 22nd, 2014	3.32	52.1	78		
Jan 27th, 2014	5.88	66.2	80		
Feb 7th, 2014	3.95	53.4	92	40	1.88

*WBC* white blood cell, *NE* neutrophil, *Hb* hemoglobin, *ESR* erythrocyte sedimentation rate, *CRP* C-reactive protein

**Fig. 6:**
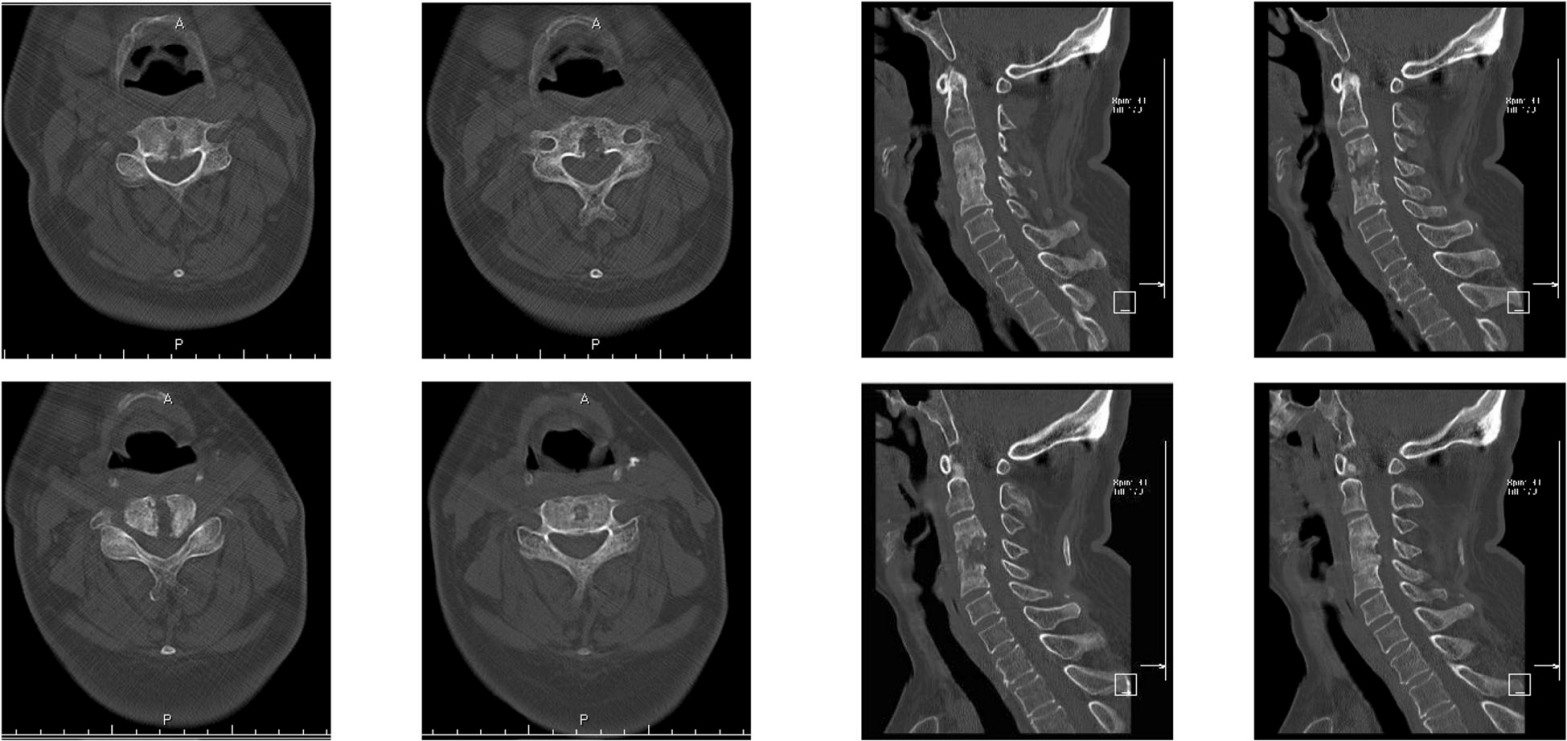
CT and MRI images 3 months after application of Hallo frame support

**Fig. 7:**
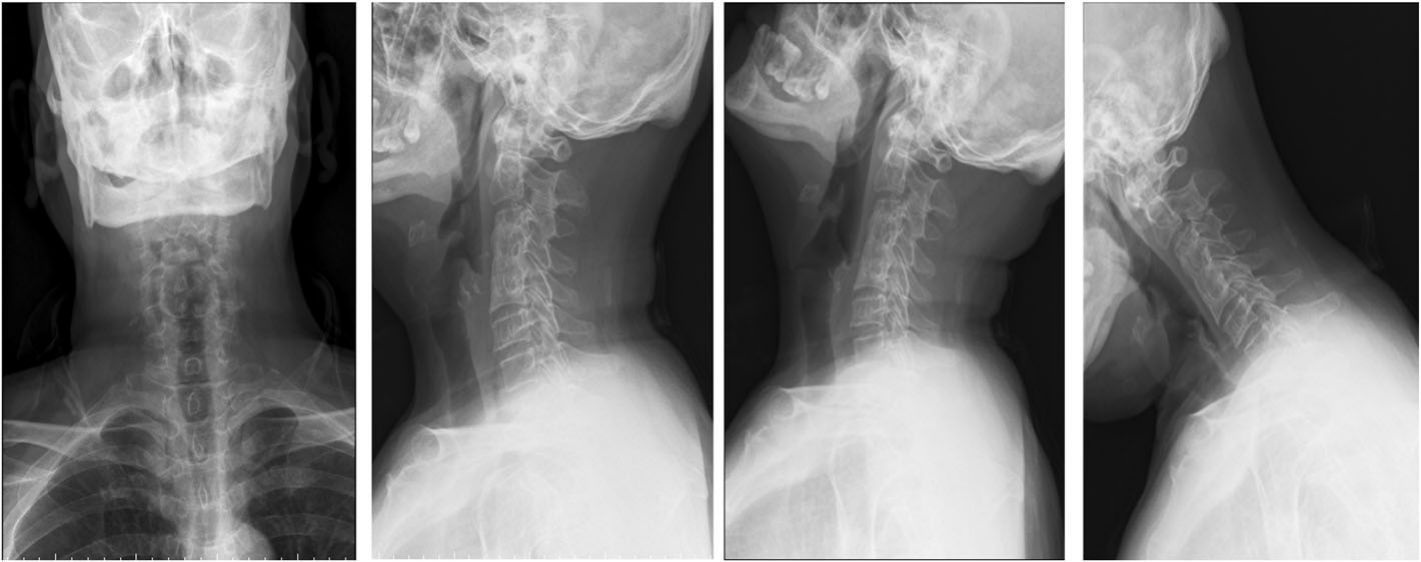
Plain radiograph 11 months after the debridement surgery

After discharge, the patient was uneventful and asymptomatic. However, neck CT scan on April 21st, 2014 (3 months after debridement and external Hallo frame application) showed incomplete fusion of cervical vertebra and the spine was unstable (Fig. 6). After continuous application of the external Hallo frame support, CT scan on May 15th, 2014 showed complete fusion of cervical vertebra and thus, the external Hallo frame was removed on May 16th, 2014. The patient was fully recovered as evidenced by the neck X-ray of 11 months after the surgery (Fig. 7).

## Discussion

Anterior cervical discectomy and fusion (ACDF) is a common procedure in cervical spine surgery for patients with degenerative cervical disc, which was first described by Cloward and Smith-Robinson [1, 18]. Recently, clinical outcomes of ACDF have been remarkably improved with improvement of implant materials and fixation techniques [8, 9, 19]. However, complications after ACDF seriously affect patients’ quality of life. In this regard, intra-operative nerve injuries (recurrent laryngeal nerve, sympathetic trunk, spinal cord contusion, nerve root injury) or vascular injuries (vertebral artery laceration, carotid artery or jugular vein injury) as well as postoperative complications (epidural and wound hematoma, aneurysm formation, wound infection, epidural abscess, and spondylodiscitis) have been reported [5, 7, 20–25].

It has been reported that the rates of variety kinds of complications associated with ACDF ranged from 0.45 to 19.8% [5, 7, 23–25]. The mortality rate was 0.1% (1 of 1015 patients, due to esophageal perforation); overall morbidity rate was 19.3% (196/1015); and the most common complication was the development of isolated postoperative dysphagia, which observed in 9.5% of our patients [5]. Wound infection rate was between 0.1 and 1.6%, and most of the infection occurred in the early postoperative period with poor wound care [5, 7, 26]. In contrast, late infection following ACDF procedure is uncommon and often associated with esophageal perforation [5, 8, 9, 27, 28].

Risk factors of deep and delayed infection after ACDF include diabetes, spinal implants of foreign body, HIV or tuberculosis infection, metabolic syndrome, and long operative time of ACDF [7, 25, 29, 30]. It has been reported that delayed esophageal perforation was associated with implant migration [5, 7, 12] or motion of the esophagus against the instrumentation [12]. The reported incidence of implant failure in ACDF was as high as 35% [7]. However, only 1% of ACDF procedures were associated with implant failure that endanger the trachea-esophageal structures [11], which was often associated with screws that were not locked [5], or prominent plate position [7]. While spontaneous infection could not be excluded in the current case, the main cause for the infection of current case could possibly be associated implant migration as shown in the neck X-ray and CT scan.

Neck pain, dysphagia, fever, and localized induration are common clinical symptoms of postoperative wound infection associated with ACDF. Previous case reports presented various complications such as elimination of screws through the mouth or the gastrointestinal tract, and extrusion of the graft and the whole fixation device [10, 26, 31, 32]. Gaudinez et al. [33] reported that 28 out of 44 patients had cervical fusion with plate and screws, and 22 cases of them had cervical osteomyelitis or neck abscess. In addition, 42 of the 44 patients required surgical repair of the esophageal injury.

Late infections after ACDF procedure may be associated with an esophageal perforation [6, 8, 10, 11]. Late infection without esophageal injury, however, has also been reported. Lu et al. [11] reported a delayed presentation of esophageal erosion 9 years after anterior cervical plate implantation. Christiano et al. [7] reported a case of late prevertebral abscess presenting with a sinus in the posterior triangle of the neck 2 years after anterior cervical fusion in the absence of esophageal perforation. Jin et al. [9] reported an extremely rare case of late infection 20 years after ACDF, who had no esophageal perforation. Consistent with these reports, the patient of current report had cervical infection over 30 days after ACDF without esophageal perforation or risk factors such as diabetes or tuberculosis. The patient, however, had implant migration, which could be possibly the cause of infection in that the presence of spinal implants as a foreign body may be a risk factor for deep and delayed wound infections [5]. While most of spinal implants used today are made of inert metals that have smooth surfaces in order to prevent bacterial adherence [16], the patient of current report was implanted titanium cage bone graft (autologous bone) at C3/4 and C4/5, which might be the cause of deep infection presented 30 days after ACDF.

Laboratory tests for diagnosis of deep infection after ACDF include complete blood cell count, ESR, CRP, neck plain radiographs, CT scan, and MRI of the neck. Ultrasonography is also often used to see the extent of abscess cavity and barium-swallow radiograph is used to detect or exclude esophageal perforation. In the current study, in addition to complete blood cell count, ESR, and CRP, neck plain radiographs, CT scan, MRI, and ultrasonography were performed for the diagnosis of abscess. Barium-swallow contrast radiograph was also conducted in order to exclude esophageal perforation.

In cases of deep cervical abscess regardless of esophageal perforation, the infecting organisms are usually those belonging to the normal bacterial flora of the pharyngo-esophageal tract, which include several types of staphylococci, streptococci, neisseriae, clostridium, and the infection is often of mixed bacteria [34]. In our patient, the causative organism was *Staphylococcus aureus*, which was positively cultured from the abscess drainage. This pathogen was resistant to penicillin G, clindamycin, erythromycin, and rifampin, but sensitive to ß-lactamase, cefoxitin, ciprofloxacin, gentamycin, vancomycin, ntrofurantoin, and levofloxacin. Therefore, in addition to surgical approach of debridement and removal of the implant, appropriate antibiotics were given based on the findings of antibiotic sensitivity assay. However, after using several kinds of antibiotics for over 3 weeks, the patient had unexpected high fever with normal white blood cell count and neutrophil count. After quitting antibiotics, patient’s body temperature returned to normal, suggesting over using of antibiotics should be avoided. In addition, the patient of current study had unstable vertebra after removal of the implant, and thus, Hallow frame was used to support healing process and to avoid additional adverse events.

In conclusion, we report a case of delayed deep cervical abscess after ACDF and cervical spine instrumentation. Late deep cervical infection should be considered if a patient had a history of ACDF and presents with dysphagia even in the absence of fever or esophageal perforation. In addition to radical debridement and removal of loosed implant, appropriate antibiotics should be used with avoiding over-usage of antibiotics. External Hallo Frame support is recommend for the patient with unstable vertebral fusion.

## Data Availability

The datasets generated and analyzed during the current study are available from the corresponding author on reasonable request.

## Abbreviations

ACDF: Anterior cervical discectomy and fusion
CT: Computerize tomography
MRI: Magnetic resonance imaging
